# [^11^C]AF150(S), an agonist PET ligand for M1 muscarinic acetylcholine receptors

**DOI:** 10.1186/2191-219X-3-19

**Published:** 2013-03-21

**Authors:** Hans JC Buiter, Albert D Windhorst, Marc C Huisman, Maqsood Yaqub, Dirk L Knol, Abraham Fisher, Adriaan A Lammertsma, Josée E Leysen

**Affiliations:** 1 Department of Nuclear Medicine & PET Research, VU University Medical Center, PO Box 7057, Amsterdam, , 1007 MB, The Netherlands; 2 Department of Epidemiology and Biostatistics, VU University Medical Center, Amsterdam, , 1007 MB, The Netherlands; 3 Israel Institute for Biological Research, Ness-Ziona, Israel

**Keywords:** PET imaging, agonist, [^11^C]AF150(S), G protein-coupled receptor, rat brain, M1 muscarinic acetylcholine receptor

## Abstract

**Background:**

The M1 muscarinic acetylcholine receptor (M1ACh-R) is a G protein-coupled receptor that can occur in interconvertible coupled and uncoupled states. It is enriched in the basal ganglia, hippocampus, olfactory bulb, and cortical areas, and plays a role in motor and cognitive functions. Muscarinic M1 agonists are potential therapeutic agents for cognitive disorders. The aim of this study was to evaluate [^11^C]AF150(S) as a putative M1ACh-R agonist PET ligand, which, owing to its agonist properties, could provide a tool to explore the active G protein-coupled receptor.

**Methods:**

Regional kinetics of [^11^C]AF150(S) in rat brain were measured using a high-resolution research tomograph, both under baseline conditions and following pre-treatment with various compounds or co-administration of non-radioactive AF150(S). Data were analysed by calculating standard uptake values and by applying the simplified reference tissue model (SRTM).

**Results:**

[^11^C]AF150(S) was rapidly taken up in the brain, followed by a rapid clearance from all brain regions. Analysis of PET data using SRTM revealed a binding potential (BP_ND_) of 0.25 for the striatum, 0.20 for the hippocampus, 0.16 for the frontal cortical area and 0.15 for the posterior cortical area, all regions rich in M1ACh-R. BP_ND_ values were significantly reduced following pre-treatment with M1ACh-R antagonists. BP_ND_ values were not affected by pre-treatment with a M3ACh-R antagonist. Moreover, BP_ND_ was significantly reduced after pre-treatment with haloperidol, a dopamine D_2_ receptor blocker that causes an increase in extracellular acetylcholine (ACh). The latter may compete with [^11^C]AF150(S) for binding to the M1ACh-R; further pharmacological agents were applied to investigate this possibility. Upon injection of the highest dose (49.1 nmol kg^−1^) of [^11^C]AF150(S) diluted with non-radioactive AF150(S), brain concentration of AF150(S) reached 100 nmol L^−1^ at peak level. At this concentration, no sign of saturation in binding to M1ACh-R was observed.

**Conclusions:**

The agonist PET ligand [^11^C]AF150(S) was rapidly taken up in the brain and showed an apparent specific M1ACh-R-related signal in brain areas that are rich in M1ACh-R. Moreover, binding of the agonist PET ligand [^11^C]AF150(S) appears to be sensitive to changes in extracellular ACh levels. Further studies are needed to evaluate the full potential of [^11^C]AF150(S) for imaging the active pool of M1ACh-R *in vivo*.

## Background

Acetylcholine (ACh) is a core neurotransmitter that controls vital functions via the peripheral nervous system (e.g. heart rate, intestinal motility, and glandular secretion) and the central nervous system (CNS) (e.g. cognitive and motor functions) [1]. Cholinergic neurotransmission is processed via nicotine and muscarinic acetylcholine receptors (MACh-R). The former are ligand-gated ion channels; the latter are metabotropic G protein-coupled receptors (GPCRs). The MACh-R family comprises five characterised subtypes (M1 to M5) [2].

M1ACh-R is the most prevalent MACh-R subtype in the CNS and acts as an excitatory receptor that is coupled to the Gα_q/11_ protein [3]. It is located primarily in postsynaptic nerve terminals in forebrain regions including the basal ganglia, hippocampus and cortical areas; the cerebellum is essentially devoid of M1ACh-R [4]. M1ACh-R in the forebrain regions play a role in motor control and cognition and is thought to be implicated in pathologies such as schizophrenia, Parkinson's disease and Alzheimer's disease [5,6]. Studies using M1ACh-R agonists in laboratory animals have shown improvement in cognitive functions [7]. Clinical studies in patients with Alzheimer's disease and schizophrenia revealed attenuation of psychotic behaviour and showed improvement of cognition [8].

A non-invasive molecular imaging technique such as positron emission tomography (PET) could be useful to elucidate the function of M1ACh-R *in vivo*. In the past, several compounds with affinity for M1ACh-R have been labelled with carbon-11, e.g. [^11^C]scopolamine, [^11^C]NMPB, [N-^11^C-methyl]-benztropine and [^11^C]QNB [9-12]. These PET ligands demonstrated good brain uptake and allowed mapping of MACh-R in primate and human brains. However, these ligands are not M1ACh-R selective, and they show slow ligand-receptor dissociation kinetics, by which ligand-receptor binding equilibrium cannot be reached *in vivo*[13,14]. In addition, the above-mentioned radioligands are antagonists that bind to the total pool of GPCRs. Consequently, they cannot distinguish between activated GPCRs and uncoupled inactive receptors. In contrast, agonists usually show high affinity for G protein-coupled receptors and low affinity for uncoupled receptors. Therefore, at low concentrations, they bind to the activated receptors only. The occurrence of interconvertible affinity states of GPCRs has been amply documented [15] and was also demonstrated for M1ACh-R in *in vitro* experiments [16,17]. Studies with agonist PET ligands for the dopamine D_2_ receptor, such as [^11^C](+)-PHNO, revealed that the agonist PET ligand labelled *in vivo* a smaller receptor pool than an antagonist PET ligand, and moreover, agonist PET ligands appeared more sensitive to displacement by released endogenous dopamine [18]. This can be taken as an indication that the agonist PET ligand preferentially labels the active G protein-coupled receptor pool, which is the target for the endogenous neurotransmitter.

Besides for the dopamine D_2_[19-22] and the μ-opiate receptor [23], few agonist PET ligands have been investigated for other GPCRs. The tested muscarinic agonists, [^11^C]xanomeline and [^11^C]butylThio-TZTP, appeared not selective for the M1ACh-R subtype and show high binding affinity for σ sites, leading to substantial non-selective binding. In addition, they suffer from poor pharmacokinetics [24].

In the search for a suitable selective M1ACh-R agonist PET ligand, AF150(S) was selected based on its pharmacological properties. AF150(S) has moderate affinity with an apparent equilibrium dissociation constant for high-affinity binding sites, *K*_d,H_ = 200 nM for M1ACh-R in rat cerebral cortex and shows functional selectivity for M1ACh-R in transfected cells [25]. AF150(S) belongs to a series of selective M1ACh-R agonists related to cevimeline (AF102B) [26]. Cevimeline, a rigid analogue of ACh, is marketed for human medicinal use and is likely to act as an orthosteric agonist. The pharmacological and potential therapeutic properties of AF150(S) have been investigated extensively in animal models of neurological diseases, where improvement in cognitive functions, reduction of amyloid plaques and decrease in tau phosphorylation were demonstrated [27-29]. AF150(S) and its congener AF267B have been considered as potential dual symptomatic and disease-modifying treatments for Alzheimer's disease [30]. Such important possible therapeutic application further supports the interest for investigating [^11^C]AF150(S) as a potential M1ACh-R agonist PET ligand.

Recently, [^11^C]AF150(S) has been synthesised successfully. Its binding properties and specificity for M1ACh-R were evaluated by *in vitro* autoradiography; high uptake in rat brain, possibly by facilitated transport mechanisms, was demonstrated in studies *ex vivo*[31].

In the present study, brain uptake and binding of [^11^C]AF150(S) were studied in rats using PET in order to explore its suitability as an agonist PET ligand for M1ACh-R. *In vivo* specificity of binding and sensitivity to changes in extracellular levels of ACh were examined by treatment with various pharmacological agents.

## Methods

### Materials

Xanomeline was obtained from Metina AB (Lund, Sweden); pirenzepine, trihexyphenidyl and haloperidol were purchased from Sigma-Aldrich (Zwijndrecht, The Netherlands); darifenacin was obtained from Sequoia Research Products Ltd. (Pangbourne, UK). AF-DX 384 was purchased from Tocris Bioscience (Bristol, UK), and rivastigmine was purchased from AvaChem Scientific LLC (San Antonio, TX, USA). Both AF400, the precursor for radiolabelling, and reference AF150(S) were provided by the Israel Institute for Biological Research, Ness-Ziona, Israel.

Compounds were dissolved in saline for *in vivo* application, except for xanomeline and haloperidol which were dissolved in ethanol and further diluted with saline (final concentration of ethanol, <10%). AF-DX 384 was dissolved in dimethyl sulfoxide (DMSO) and diluted with saline (final concentration of DMSO, <5%).

Cold (non-radiolabelled) AF150(S) was dissolved in saline to obtain a 10-mmol L^−1^ stock solution, from which further dilutions were made. Concentrations of AF150(S) in all solutions were verified by HPLC.

### Animals

Experiments were performed with male Wistar rats (304 ± 43 g; Harlan Netherlands B.V. Horst, The Netherlands). The rats were kept in conditioned housing under a regular light/dark cycle (12/12 h) and allowed food and water *ad libitum*. All animal experiments were in compliance with Dutch law and approved by the VU University Animal Ethics Committee.

### Radiochemistry

[^11^C]AF150(S) was synthesised as previously described by methylation of the desmethyl precursor (AF400) using [^11^C]methyl iodide [31]. The final product had a radiochemical purity >99% and a specific activity (SA) of 23 to 118 GBq μmol^−1^ at the end of synthesis. [^18^F]NaF was prepared as described previously [32]. Both radiotracers were formulated in an isotonic, sterile and pyrogen-free solution for intravenous (IV) injection.

### PET studies

#### PET scanner

PET measurements were performed using an ECAT high-resolution research tomograph (HRRT) (CTI/Siemens, Knoxville, TN, USA). The ECAT HRRT is a dedicated human brain PET scanner, with design features that enable high spatial resolution combined with high sensitivity, making it also suitable for small-animal imaging. This scanner has a field of view of 312 and 250 mm in transaxial and axial directions, respectively. The spatial resolution ranges from 2.3 to 3.2 mm full width at half maximum (FWHM) in the transaxial direction and from 2.5 to 3.4 mm FWHM in the axial direction, depending on the distance from the centre as described previously [33].

#### Scan protocol

Anaesthesia was induced and maintained by constant insufflation of 1.5% to 2% isoflurane in pure oxygen. Animals were placed in a fixation device with a tooth bar to secure a fixed and immobile horizontal position of the head during scanning. Body temperature was kept constant at 37°C with a heating pad coupled to a thermostat, which was connected to a rectal thermometer. A cannula was inserted into the vena femoralis for radiotracer injection. Transmission measurements of 6-min duration were performed using a 740-MBq 2D fan-collimated ^137^Cs moving point source.

An IV bolus of 15.5 ± 4.2 MBq of [^11^C]AF150(S) was injected. At the first injection for baseline scanning, the SA was 71 ± 16 GBq μmol^−1^; at the second injection, following pre-treatment, SA was 9 ± 2 GBq μmol^−1^. A three-dimensional (3D) emission scan was acquired, starting immediately prior to the IV injection and lasting for 45 min. At the end of the [^11^C]AF150(S) scans, [^18^F]NaF (15.2 ± 4.5 MBq) was injected, and 30 min later, an emission scan was acquired for 30 min.

#### Image reconstruction

Acquired PET data were stored in 64-bit list mode format and, for [^11^C]AF150(S), were subsequently histogrammed into 21 frames with progressive duration (7 × 10, 1 × 20, 2 × 30, 2 × 60, 2 × 150 and 7 × 300 s). The [^18^F]NaF data were histogrammed into a single frame of 30 min. Data were reconstructed using 3D ordered subsets weighted least squares [34] using seven iterations and 16 subsets. All data were normalised and corrected for attenuation, randoms, scatter, decay and dead time. All images were reconstructed into a matrix of 256 × 256 × 207 voxels with a voxel size of 1.218 × 1.218 × 1.218 mm^3^.

#### Pre-treatment with muscarinic agents

A total of 20 rats were used to examine the binding specificity of [^11^C]AF150(S) in the brain. Rats were randomised into five groups of four rats, and for each rat, two consecutive [^11^C]AF150(S) scans were acquired with a 30-min interval. The first scan was performed under baseline conditions, providing information on regional distribution and kinetics of [^11^C]AF150(S) in the brain. The second scan was performed following pre-treatment with either the M4/M1ACh-R agonist xanomeline (5 or 30 mg kg^−1^ subcutaneous (SC)) [35], the M1ACh-R antagonists pirenzepine (30 mg kg^−1^ SC) [36] or trihexyphenidyl (3 mg kg^−1^ SC) [37], or the M3ACh-R antagonist darifenacin (3 mg kg^−1^ IV) [38]. Xanomeline, pirenzepine and trihexyphenidyl were administered 30 min and darifenacin 15 min prior to [^11^C]AF150(S) injection.

#### Pre-treatment with agents affecting extracellular ACh levels

Twelve rats were used to examine indirect effects of haloperidol (a dopamine D_2_ antagonist and σ site ligand) [39] and AF-DX 384 (an M2/M4ACh-R antagonist) [40], the latter with and without the acetylcholine esterase (AChE) inhibitor rivastigmine [41], on [^11^C]AF150(S) binding. Rats were randomised into three groups of four rats. In each animal, two consecutive scans were performed with a 45-min interval. The first scan was performed under baseline conditions, and the second after pre-treatment with haloperidol (1 mg kg^−1^ SC), the combination of AF-DX 384 (5 mg kg^−1^ intraperitoneal (IP)) and rivastigmine (2.5 mg kg^−1^ SC), or AF-DX 384 (5 mg kg^−1^ IP). Haloperidol and AF-DX 384 were administered 30 min and rivastigmine 45 min prior to the [^11^C]AF150(S) injection.

#### Co-injection of [^11^C]AF150(S) with cold AF150(S)

Twelve rats were used to investigate the effect of co-injection of cold AF150(S) on the total amount of AF150(S) taken up into the brain. Cold AF150(S) (1, 5 and 15 nmol, respectively) was added to the dose of [^11^C]AF150(S) in the syringe, right before intravenous injection. Rats were randomised into three groups of four animals; each animal underwent two consecutive scans with a 30-min interval. First, each rat received an injection of undiluted [^11^C]AF150(S) to register a baseline scan; before the second scan, rats received an injection of [^11^C]AF150(S) diluted with cold AF150(S).

### Data analysis

#### Region of interest analysis

[^18^F]NaF scans were co-registered with a standard magnetic resonance (MR) rat brain template to delineate regions of interest (ROIs); this method was previously described and validated with [^11^C]AF150(S) in rats [42]. The following ROIs were used: left striatum (15 mm^3^), right striatum (15 mm^3^), hippocampus (left and right together, 27 mm^3^), frontal plus parietal cortical area (49 mm^3^), posterior plus occipital cortical area (49 mm^3^) and cerebellum (86 mm^3^). ROIs were transferred onto dynamic PET images in order to calculate regional radioactivity concentrations (kBq mL^−1^) and to generate regional time-activity curves (TACs). TACs were also normalised for generation of standardised uptake values (SUVs) [43].

#### Binding potential

The simplified reference tissue model (SRTM) [44], with cerebellum as reference tissue, was used to calculate non-displaceable binding potential (BP_ND_) as an outcome measure of specific binding.

### Statistical analysis

Differences in regional BP_ND_ values at baseline were assessed using one-way ANOVA with Bonferroni correction, and differences in BP_ND_ values before and after treatment with various agents or co-administration of non-radioactive AF150(S) were tested using a general Linear Mixed Model (SPSS Statistics version 17.0; SPSS Inc., Chicago, IL, USA). All results are expressed as mean ± standard deviation (SD), and values of *p* < 0.05 were considered to be statistically significant.

## Results

### Baseline uptake

**Figure 1:**
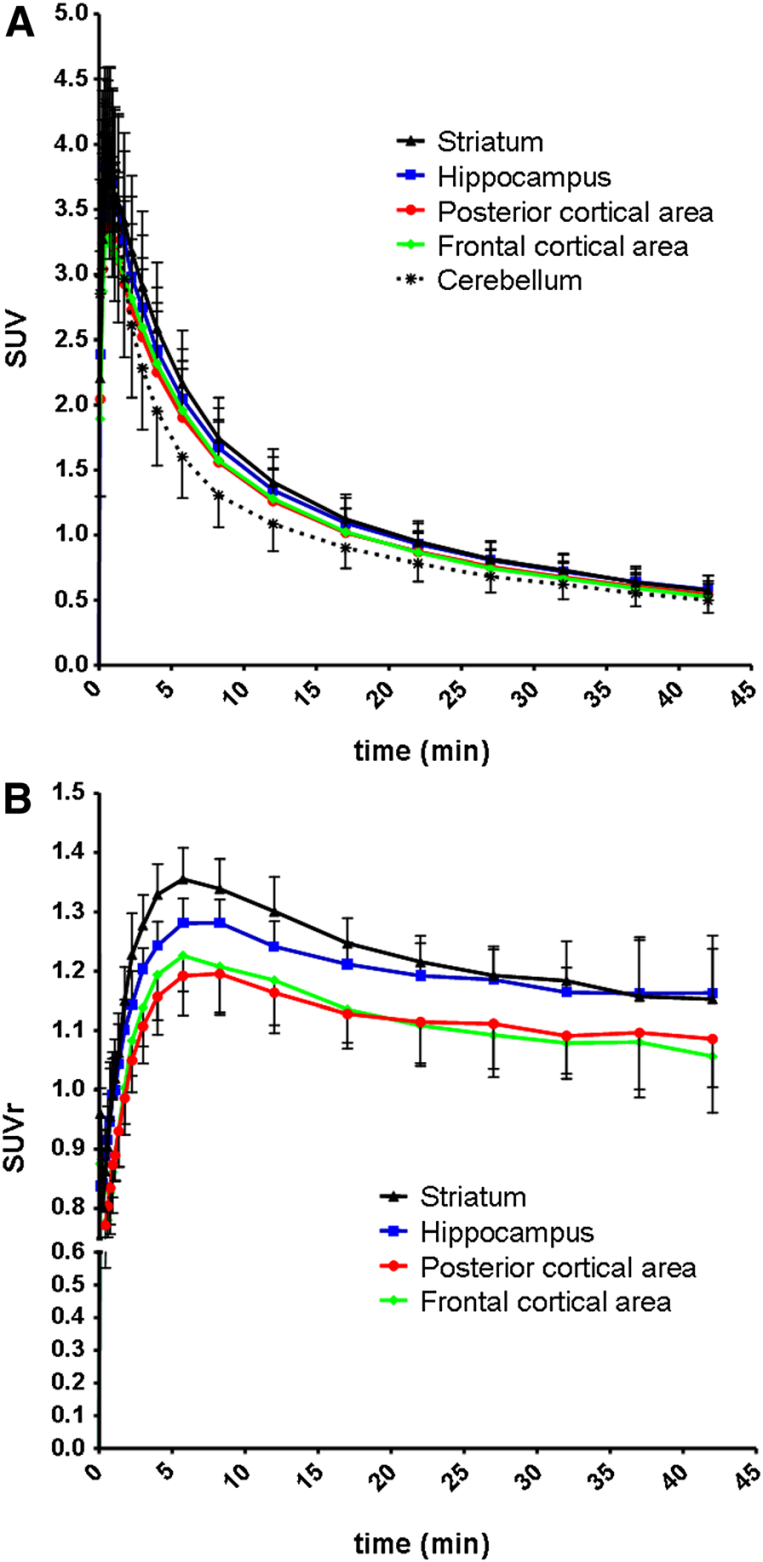
**Standard uptake values of [**^**11**^**C]AF150(S) in various brain regions obtained from PET experiments.** (**A**) Regional brain concentration, expressed as SUV, as a function of time following IV injection of [^11^C]AF150(S) and (**B**) relative to cerebellar concentration (SUVr). Each data point represents the mean of 44 animals. Vertical lines indicate the SD.

[^11^C]AF150(S) uptake in brain regions under baseline conditions was examined in a total of 44 rats. Average time-activity curves for five brain regions are shown in Figure 1A. Following intravenous injection of [^11^C]AF150(S), radioactivity in all brain regions rapidly increased and reached its maximum within the first minute after injection. Uptake in the striatum, hippocampus and the two cortical areas was higher than in the cerebellum. Clearance of radioactivity from the brain was relatively fast, especially in the cerebellum. The maximum brain concentration measured was, on average, 155 ± 26 kBq mL^−1^ corresponding to 2.9 ± 0.5 nM [^11^C]AF150(S).

SUV ratios (SUVr) of the brain region over the cerebellum as a function of time are shown in Figure 1B. SUVr values were maximal at 5.8 min, ranging from 1.19 ± 0.07 for the posterior cortical area to 1.35 ± 0.05 for the striatum.

**Figure 2:**
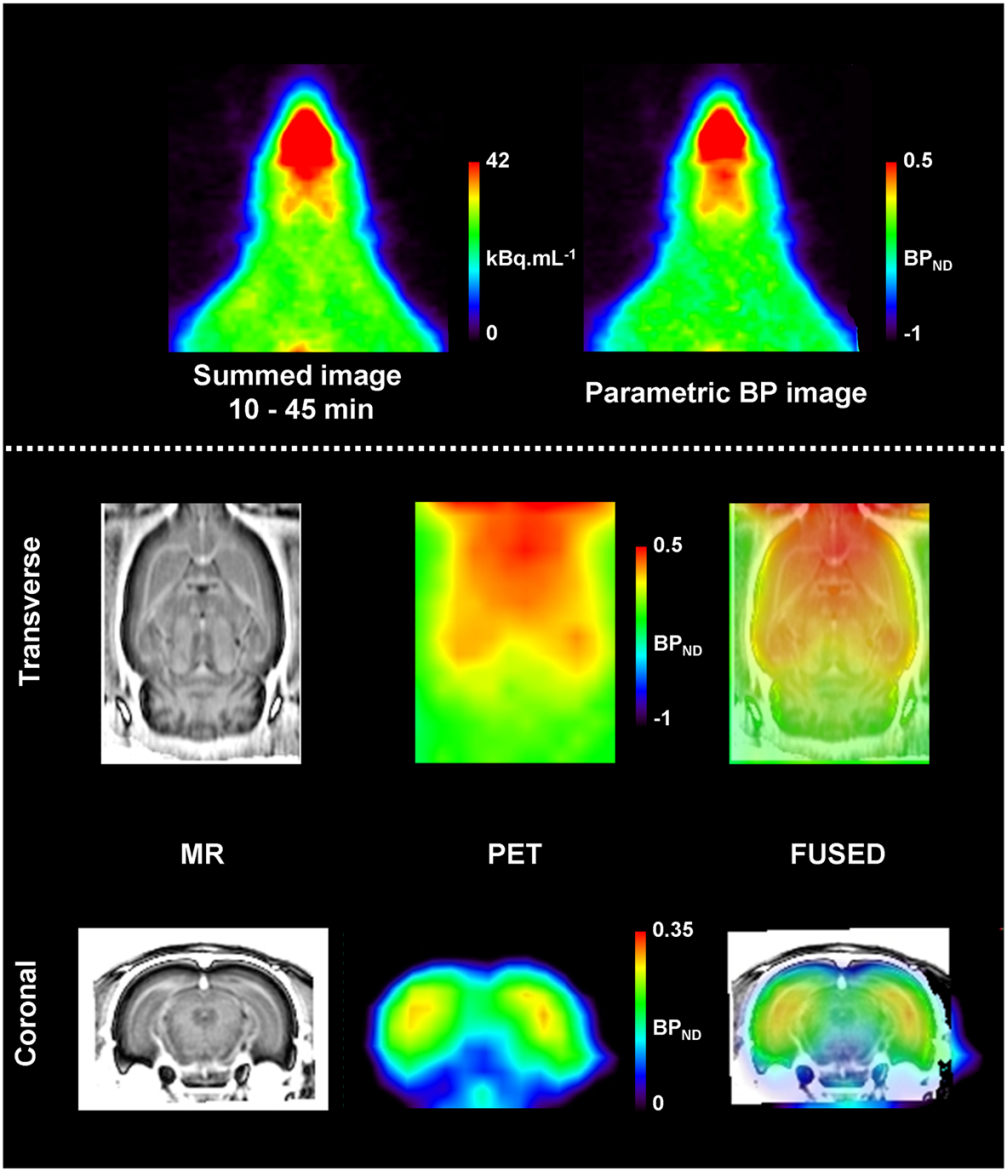
**Example of a [**^**11**^**C]AF150(S) PET image of the brain.** Summed (10 to 45 min after injection) baseline [^11^C]AF150(S) uptake image together with corresponding parametric non-displaceable binding potential (BP_ND_) image. Corresponding MR slices and fused images are shown for anatomical reference.

[^11^C]AF150(S) specific binding in M1ACh-R-rich brain areas was determined using SRTM with the cerebellum as reference tissue. BP_ND_ values are presented in Table 1. BP_ND_ under baseline conditions ranged from 0.25 to 0.15, with a regional rank order of striatum > hippocampus > frontal cortical area ≈ posterior cortical area. Findings were quite consistent across the five groups of four rats. Representative images of baseline [^11^C]AF150(S) uptake and corresponding parametric BP_ND_ are shown in Figure 2.

**Table 1:** **BP**_**ND**_**of [**^**11**^**C]AF150(S) before and after treatment with various muscarinic agents**

**Brain region**	**Xanomeline**		**Xanomeline**		**Pirenzepine**		**Trihexyphenidyl**		**Darifenacin**		**Mean**
	**(5 mg kg**^**−1**^**SC)**		**(30 mg kg**^**−1**^**SC)**		**(30 mg kg**^**−1**^**SC)**		**(3 mg kg**^**−1**^**SC)**		**(3 mg kg**^**−1**^**IV)**		**(*n*= 20)**
	**Baseline**	**Pre-treated**	**Baseline**	**Pre-treated**	**Baseline**	**Pre-treated**	**Baseline**	**Pre-treated**	**Baseline**	**Pre-treated**	**Baseline**
Left striatum	0.25 ± 0.05	0.30 ± 0.04^*^	0.22 ± 0.07	0.22 ± 0.05	0.25 ± 0.04	0.21 ± 0.05^*^	0.24 ± 0.02	0.21 ± 0.01	0.29 ± 0.03	0.28 ± 0.04	0.25 ± 0.05^a^
Right striatum	0.24 ± 0.04	0.27 ± 0.01	0.23 ± 0.08	0.25 ± 0.06	0.25 ± 0.06	0.23 ± 0.03	0.22 ± 0.01	0.22 ± 0.01	0.26 ± 0.05	0.26 ± 0.03	0.24 ± 0.05^b^
Hippocampus	0.20 ± 0.03	0.24 ± 0.01^*^	0.19 ± 0.04	0.19 ± 0.06	0.20 ± 0.03	0.18 ± 0.02	0.19 ± 0.01	0.16 ± 0.01^**^	0.24 ± 0.02	0.22 ± 0.04	0.20 ± 0.03^c^
Frontal cortical area	0.12 ± 0.07	0.10 ± 0.04	0.17 ± 0.06	0.14 ± 0.04	0.16 ± 0.05	0.10 ± 0.04^**^	0.11 ± 0.03	0.05 ± 0.03^*^	0.22 ± 0.02	0.20 ± 0.06	0.16 ± 0.06^d^
Posterior cortical area	0.12 ± 0.06	0.10 ± 0.04	0.15 ± 0.06	0.13 ± 0.04	0.13 ± 0.03	0.09 ± 0.03^**^	0.11 ± 0.05	0.05 ± 0.03^*^	0.23 ± 0.01	0.18 ± 0.05	0.15 ± 0.06^e^

Data are presented as mean ± SD (*n* = 4 per group). IV, intravenous; SC, subcutaneous. Significant difference compared with baseline: ^*^p < 0.05, ^**^p < 0.01. Significantly different, with *p* < 0.001: ^a^all regions (except for right striatum), ^b^all regions (except for left striatum), ^c^compared to all other brain regions, ^d^all regions (except for the posterior cortical area), or ^e^all regions (except for the frontal cortical area).

### Pre-treatment with muscarinic agents

In Table 1, the BP_ND_ values in five brain regions following pre-treatment with xanomeline, pirenzepine, trihexyphenidyl and darifenacin are shown. Following pre-treatment with xanomeline at 5 mg kg^−1^, [^11^C]AF150(S) BP_ND_ significantly increased by 20% and 19% in the left striatum and hippocampus, respectively. Xanomeline at a higher dose (30 mg kg^−1^) did not result in significant changes in BP_ND_ in any of the brain areas investigated. This high dose caused severe side effects, such as strongly increased heart rate, salivation and irregular breathing. Pre-treatment with pirenzepine and trihexyphenidyl caused significant reductions in BP_ND_. With pirenzepine, reductions of 37%, 32% and 16% were seen in the frontal cortical area, posterior cortical area and left striatum, respectively. Trihexyphenidyl caused reductions in the frontal cortical area, posterior cortical area and hippocampus of 53%, 56% and 13%, respectively. Pre-treatment with darifenacin had no measurable effect on [^11^C]AF150(S) binding in any brain region.

### Pre-treatment with agents affecting extracellular ACh levels

Table 2 provides BP_ND_ values in five brain areas following pre-treatment with haloperidol, AF-DX 384 and AF-DX 384 plus rivastigmine. Haloperidol pre-treatment resulted in significant reductions in BP_ND_ in the right striatum and hippocampus of 27% and 15%, respectively. The animals showed severe rigidity following the haloperidol treatment. Pre-treatment with AF-DX 384, however, did not show any significant changes in BP_ND_. The addition of rivastigmine to AF-DX 384 pre-treatment resulted in significant increases in BP_ND_ of [^11^C]AF150(S) in the right striatum, hippocampus and frontal cortical area of 22%, 12% and 24%, respectively. These treatments did not have an apparent effect on the animal's behaviour or body posture.

**Table 2:** **BP**_**ND**_**of [**^**11**^**C]AF150(S) without and with pre-treatment with agents that increase extracellular levels of ACh**

**Brain region**	**Haloperidol**		**AF-DX 384**		**AF-DX 384 and rivastigmine**	
	**(1 mg kg**^**−1**^**SC)**		**(5 mg kg**^**−1**^**IP)**		**(5 mg kg**^**−1**^**IP and 2.5 mg kg**^**−1**^**SC)**	
	**Baseline**	**Pre-treated**	**Baseline**	**Pre-treated**	**Baseline**	**Pre-treated**
Left striatum	0.23 ± 0.04	0.18 ± 0.01	0.27 ± 0.06	0.32 ± 0.04	0.25 ± 0.05	0.27 ± 0.03
Right striatum	0.28 ± 0.05	0.21 ± 0.03^*^	0.30 ± 0.04	0.33 ± 0.05	0.23 ± 0.05	0.29 ± 0.03^*^
Hippocampus	0.19 ± 0.02	0.16 ± 0.01^*^	0.26 ± 0.04	0.26 ± 0.02	0.22 ± 0.02	0.25 ± 0.01^*^
Frontal cortical area	0.10 ± 0.06	0.06 ± 0.04	0.24 ± 0.02	0.24 ± 0.06	0.17 ± 0.03	0.21 ± 0.02^**^
Posterior cortical area	0.07 ± 0.07	0.06 ± 0.04	0.21 ± 0.03	0.22 ± 0.05	0.19 ± 0.04	0.19 ± 0.04

Data are presented as mean ± SD (*n* = 4 per group). SC, subcutaneous; IP, intraperitoneal. Significant difference compared with baseline: ^*^p < 0.05, ^**^p < 0.01.

### Co-injection with cold AF150(S)

**Figure 3:**
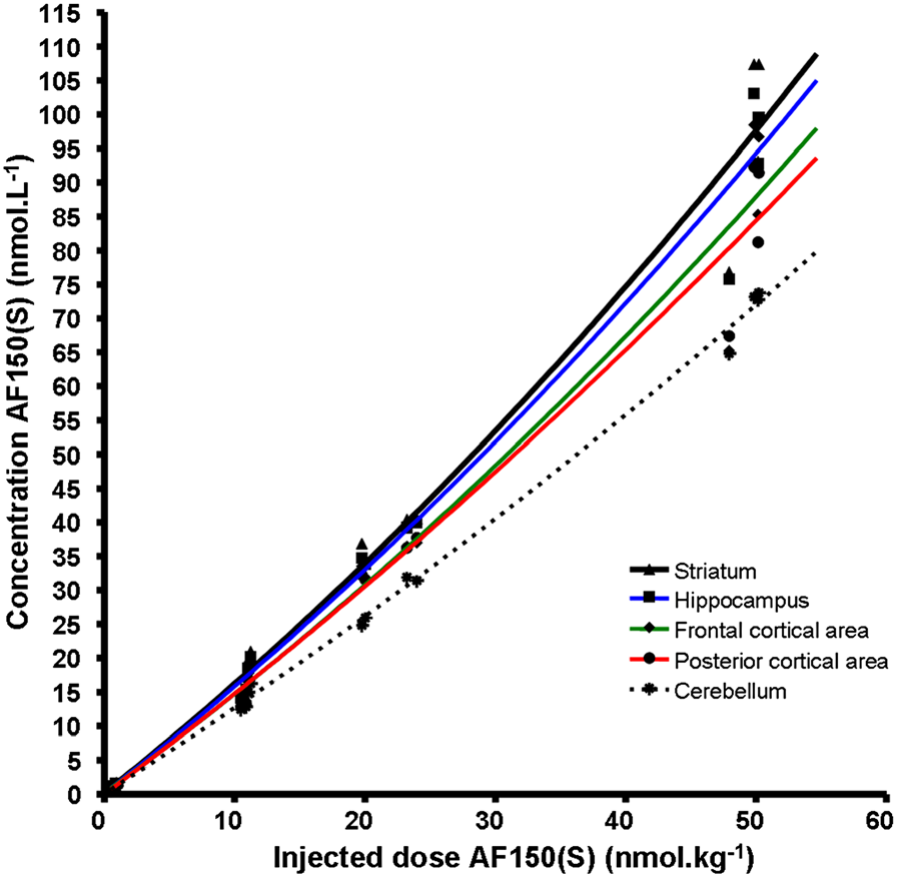
**Effect of co-injection of cold AF150(S) on regional brain uptake of AF150(S) in various brain regions.** Concentrations of cold AF150(S) in those brain areas were calculated based on measured radioactivity concentrations of [^11^C]AF150(S) at 5.8 min and the specific activity at the time of injection. Solid lines are the non-linear fits of the data using GraphPad Prism (version 4.00, San Diego, CA, USA). Possible metabolism of AF150(S) was not taken into account.

In Table 3, doses of co-injected cold AF150(S) and resulting SA of injected [^11^C]AF150(S) are shown. The aim of the experiment was to explore whether an AF150(S) concentration occupancy curve could be achieved. Effects of co-injection of cold AF150(S) on the regional brain uptake of total AF150(S) (radioactive + cold calculated according to the SA of the injected sample), measured at 5.8 min after injection, are shown in Figure 3. The increase in brain concentration of total AF150(S) was larger when based on a linear relationship between injected dose and brain concentration. Possible metabolism of AF150(S) was not taken into account. Calculated BP_ND_ values under baseline conditions and after co-administration of cold AF150(S) were not significantly different, see Table 4. Hence, a saturation curve of specific binding of AF150(S) could not be constructed.

**Table 3:** **Overview of injected doses of [**^**11**^**C]AF150(S) and AF150(S)**

**Cold AF150(S) co-injected per rat (nmol)**	**Number**	**[**^**11**^**C]AF150(S) injected radioactivity (MBq)**	**Specific activity of cold diluted [**^**11**^**C]AF150(S) at injection (GBq μmol**^**−1**^**)**	**AF150(S) total dose (nmol kg**^**−1**^**)**
0	12	16.9 ± 0.8	82.9 ± 8.4	0.7 ± 0.1
1	4	17.8 ± 0.3	6.4 ± 0.0	10.9 ± 0.3
5	4	15.7 ± 2.1	2.4 ± 0.3	21.7 ± 2.2
15	4	18.2 ± 1.1	1.1 ± 0.1	49.1 ± 1.2

**Table 4:** **BP**_**ND**_**of [**^**11**^**C]AF150(S) without and with co-injection of cold AF150(S)**

**Brain region**	**+1 nmol of cold AF150(S)**		**+5 nmol of cold AF150(S)**		**+15 nmol of cold AF150(S)**	
	**Baseline**	**Co-injected**	**Baseline**	**Co-injected**	**Baseline**	**Co-injected**
Left striatum	0.23 ± 0.02	0.21 ± 0.02	0.26 ± 0.06	0.25 ± 0.09	0.28 ± 0.08	0.31 ± 0.11
Right striatum	0.24 ± 0.01	0.23 ± 0.02	0.26 ± 0.06	0.26 ± 0.09	0.30 ± 0.07	0.32 ± 0.11
Hippocampus	0.20 ± 0.01	0.18 ± 0.03	0.23 ± 0.04	0.23 ± 0.07	0.24 ± 0.04	0.26 ± 0.08
Frontal cortical area	0.11 ± 0.04	0.08 ± 0.03	0.16 ± 0.06	0.14 ± 0.09	0.17 ± 0.09	0.18 ± 0.12
Posterior cortical area	0.08 ± 0.05	0.06 ± 0.04	0.16 ± 0.05	0.13 ± 0.07	0.14 ± 0.07	0.13 ± 0.09

Statistical analysis: significant difference between baseline and co-injected, *p* ≥ 0.12.

## Discussion

### [^11^C]AF150(S) brain uptake under baseline conditions

Under baseline conditions, brain kinetics of [^11^C]AF150(S) were very rapid with peak values being reached within 1 min after injection, followed by a rapid decline. The calculated maximal brain concentration of [^11^C]AF150(S) of 2.9 nM remained approximately 70 times below the *K*_d,H_ (200 nM), of AF150(S) for M1ACh-R, under baseline conditions. In consecutive scans (baseline and following pre-treatment), the same synthesis batch of [^11^C]AF150(S) was used. As a consequence, the SA was about eight times lower at the second injection, and to keep the injected amount of radioactivity constant, a larger dose of AF150(S) was injected.

SUVr data showed increased retention in M1ACh-R-rich areas. The rank order of uptake, striatum ≥ hippocampus > frontal cortical area > posterior cortical area >> cerebellum, corresponds with the rank order of M1ACh-R density measured *in vitro* in rat brain [4]. These findings also confirm previous biodistribution data with [^11^C]AF150(S) uptake measured *ex vivo*[31]. Note that the parametric image under baseline conditions clearly shows [^11^C]AF150(S) uptake in the hippocampus (Figure 2). BP_ND_ values could successfully be derived by applying SRTM referring to the cerebellum, a region which is essentially devoid of M1ACh-R [4]. This particular reference model best fitted the [^11^C]AF150(S) PET data. Furthermore, a reference tissue model was chosen for data analysis as no arterial input function could readily be obtained from rats.

### Pre-treatment with muscarinic agents

Pre-treatment conditions, i.e. time of injection and dose of the applied compounds, were chosen according to their maximal pharmacological activity in rats. Pre-treatment with the M1ACh-R selective antagonists pirenzepine and trihexyphenidyl caused a decrease in BP_ND_ of [^11^C]AF150(S) in the frontal and posterior cortical areas. In addition, pirenzepine caused a significant reduction in BP_ND_ in the left striatum, and trihexyphenidyl in the hippocampus, indicative of a reduction in specific binding to M1ACh-R in these regions.

The M4/M1ACh-R agonist xanomeline, selected to investigate agonist-agonist competition, at a dose of 5 mg kg^−1^ caused an increase in BP_ND_ in the striatum and hippocampus, whereas a decrease would have been expected if [^11^C]AF150(S) and xanomeline are competing for the same binding site. Since GPCRs are dynamic structures that undergo configurational changes upon agonist binding, several explanations are possible for this apparent anomalous finding. Xanomeline is reported to induce M1ACh-R activation via both orthosteric and ectopic binding sites [45,46]. Xanomeline-induced activation of the ectopic site could cause increased M1ACh-R G protein coupling by which more activated orthosteric sites become available for [^11^C]AF150(S) binding, leading to higher BP_ND_ of [^11^C]AF150(S). An alternative explanation could be the effect of xanomeline on M4ACh-R. M4ACh-R is an autoreceptor, predominantly present in the striatum and hippocampus [47]. Agonist stimulation of M4ACh-R leads to a decrease in acetylcholine release [48], potentially resulting in reduced competition between endogenous extracellular acetylcholine and [^11^C]AF150(S), which in turn may result in increased binding of [^11^C]AF150(S). However, a higher dose of xanomeline (30 mg kg^−1^) that caused severe peripheral and cardiac effects, and glandular secretion, did not show any significant effect on BP_ND_ of [^11^C]AF150(S) in M1ACh-R-rich areas. Therefore, the observed effects of xanomeline seem to be dose dependent, and pharmacokinetic effects may play a role.

*In vitro* experiments have demonstrated partial agonist/antagonist effects of AF150(S) on M3ACh-R (Fisher et al., 2007, unpublished results). M3ACh-R is present in glands and can also be found on smooth muscles of blood vessels and at low density in the brain [49]. Darifenacin, an M3 antagonist with central activity, was selected to check for possible labelling of M3ACh-R by [^11^C]AF150(S). Pre-treatment with darifenacin, however, did not significantly reduce BP_ND_ of [^11^C]AF150(S) in any brain region. Hence, [^11^C]AF150(S) appears not to show measurable binding to M3ACh-R in the brain *in vivo*.

### Pre-treatment with agents affecting extracellular ACh levels

Pre-treatment conditions, i.e. time of injection and dosing, were chosen to give maximal ACh release at 5.8 min post intravenous injection of [^11^C]AF150(S) in order to have maximal competition. Haloperidol pre-treatment causes blockade of D_2_ receptors, which in turn leads to reduced inhibition of the cholinergic system, in particular in the striatum and hippocampus. This results in increased extracellular levels of acetylcholine [50]. Increase in ACh release following haloperidol treatment is demonstrated in *in vivo* microdialysis studies [51]. Further evidence for increased cholinergic activity is apparent in behavioural studies where haloperidol causes severe catalepsy as a result of cholinergic over-activity in the striatum [52]. Also in this study, at the applied dose, haloperidol caused immobility and severe rigidity in the animals. The significant reductions in BP_ND_ of [^11^C]AF150(S) in the right striatum (−27%) and hippocampus (−15%) following haloperidol treatment can likely be ascribed to increased extracellular ACh levels, which may have competed with [^11^C]AF150(S) for M1ACh-R binding in these particular brain areas.

AF-DX 384 is an M2/M4 selective muscarinic antagonist that acts on M2 autoreceptors located presynaptically on cholinergic nerve terminals. Blockade of the M2 autoreceptor will cause increased release of acetylcholine [53], which could cause competition between ACh and [^11^C]AF150(S) for the M1ACh-R, located postsynaptically. In microdialysis studies in rats, AF-DX 384 given IP at a dose of 5 mg kg^−1^ resulted in a long-lasting significant increase in acetylcholine release, 30 min after administration, in both the cortex and hippocampus [40]. In those studies, breakdown of extracellular ACh was prevented by adding the AChE inhibitor physostigmine in the microdialysis perfusate. In the present study, AChE inhibition as protection against ACh breakdown was attempted by using co-treatment with rivastigmine [41]. AF-DX 384 and rivastigmine, at the present dose, did not cause animal immobility or catalepsy, and such effect on behaviour by AF-DX 384 treatment has neither been reported in the literature. Therefore, the effect-size of M2ACh-R blockade on acetylcholine release probably is much less pronounced than that of haloperidol. Pre-treatment with only AF-DX 384 also did not have an effect on BP_ND_ in M1ACh-R-rich brain areas.

Pre-treatment with AF-DX 384 combined with rivastigmine, surprisingly, resulted in significant increases in BP_ND_ of [^11^C]AF150(S) in M1ACh-R-rich brain areas, e.g. the right striatum, hippocampus and frontal cortical area. A tentative explanation for these observed increases in BP_ND_ could be agonist-mediated changes in M1ACh-R affinity/availability. This has been proposed for the dopamine D_2_ receptor radioligand [^11^C]raclopride which demonstrated increased BP after pre-treatment with l-dopa [54]. Unfortunately, experimental evidence to explain such phenomena is hard to obtain. Nevertheless, [^11^C]AF150 binding appears to be sensitive to changes in extracellular ACh levels, in particular when behavioural effects are apparent. Ideally, and in analogy with the dopamine D_2_ agonist PET ligand studies, the sensitivity of [^11^C]AF150(S) to changes in extracellular ACh should be assessed in direct comparison with an M1ACh-R antagonist PET ligand to investigate difference in sensitivity between them. Indeed for the dopamine D_2_ agonist PET ligands [^11^C]MNPA, [^11^C]NPA and [^11^C](+)-PHNO, a larger reduction in binding, by increased endogenous dopamine levels following amphetamine pre-treatment, was found as compared to the antagonist PET ligand [^11^C]raclopride [18,55-58]. This can be taken as an indication that the agonist PET ligands preferentially label the G protein-coupled receptors that are the target of the endogenous neurotransmitter. The present observations warrant further studies, preferentially in larger animals, to investigate and further characterise the sensitivity of specific [^11^C]AF150(S) binding in brain regions to alterations in extracellular ACh levels.

### Co-injection of [^11^C]AF150(S) with cold AF150(S)

Increasing amounts of co-injected cold AF150(S) resulted in a more-than linear increase in corresponding brain concentrations of total AF150(S) in M1ACh-R-rich brain areas (Figure 3), whereas in M1ACh-R-poor brain areas, such as the cerebellum, this effect was less pronounced. After co-administration of the highest dose of cold AF150(S), BP_ND_ values were slightly increased compared to baseline conditions, yet due to variability, these values were statistically not significantly different (Table 4). The more-than-linear increase in brain concentrations of [^11^C]AF150(S) in M1ACh-R-rich brain areas is probably not due to diffusion but is a result of facilitated transport of AF150(S) in the brain that was hypothesised from analyses of results in our previous study of [^11^C]AF150(S) uptake into the brain *ex vivo*[31]. The hypothesis was mainly based on (1) the measured low lipophilicity of [^11^C]AF150(S), logD_pH 7.4_ = 0.05, which hampers diffusion through lipid cell membranes, (2) the eight-times-higher brain level as compared to plasma levels of [^11^C]AF150(S) and (3) the structural similarity between AF150(S) and nicotine, for which facilitated transport into the brain has been demonstrated.

The highest administered molar dose of non-radiolabelled AF150(S) (approximately 50 nmol kg^−1^) resulted in a brain concentration of approximately 100 nM AF150(S) *in vivo*, a value that still lies 50% under the *in vitro K*_d,H_ of AF150(S) (200 nM). At the highest applied dose, no sign of saturation in binding to M1ACh-R was observed; indeed, the latter is only expected to occur at a concentration of 4× *K*_d,H_. Therefore, saturation should be investigated using a substantial higher dose of non-radiolabelled AF150(S).

### Further evaluation of [^11^C]AF150(S) as a potential agonist PET ligand for M1ACh-R

The findings in this preliminary PET study with [^11^C]AF150(S) in rat are indicative of the potential of [^11^C]AF150(S) for specific labelling of M1ACh-R. However, PET studies in rat suffer from several limitations such as the difficulty of getting an arterial input function, the small brain regions and the resolution of the PET camera. Therefore, [^11^C]AF150(S) should be further explored in PET studies in primates and humans. The chances of success and merit of such studies are supported by the following. M1ACh-R density in primate and human brain is about 50% higher than in rat brain, according to *in vitro* autoradiography studies [16]. No species differences in the binding affinity of compounds for M1ACh-R between various species including rat, primate and human have become apparent [59]; in this respect, it was found that the potency of AF150(S) to stimulate human M1ACh-R expressed in cells fully matches its binding affinity for M1ACh-R in rat brain homogenates (personal observations, manuscript in preparation). AF150(S) shows a beneficial safety profile, and close congeners of AF150(S) have been in clinical studies and are being investigated as potential treatment (symptomatic and disease modification) for Alzheimer's disease [30].

## Conclusions

The agonist radioligand [^11^C]AF150(S) was rapidly taken up in the brain and showed fast kinetics with a significant M1ACh-R-related signal in brain areas that are rich in M1ACh-R. Moreover, binding of the agonist PET ligand [^11^C]AF150(S) appears to be sensitive to changes in extracellular ACh levels. Further preclinical and clinical studies are needed to evaluate the full potential of [^11^C]AF150(S) for imaging the active pool of M1ACh-R *in vivo*.

## Competing interests

The authors declare that they have no competing interests.

## Authors’ contributions

HJCB performed the carbon-11 tracer synthesis and all animal studies. Data analysis and modelling were performed by HJCB, MCH and MY. Statistical analysis of the outcome measures was performed by HJCB and DLK. HJCB, ADW, MCH and JEL participated in the study design and discussion and interpretation of findings. HJCB drafted the manuscript. ADW, MCH, AF, JEL, and AAL revised and gave advice for improvement of the manuscript. All authors read and approved the final manuscript.
